# Electrocardiographic Abnormalities of Takotsubo Cardiomyopathy in a Patient with Paced Ventricular Rhythm

**DOI:** 10.4061/2010/643832

**Published:** 2010-06-08

**Authors:** Krati Chauhan, Siva P. Sontineni, Venkata M. Alla, Mark J. Holmberg

**Affiliations:** ^1^Department of Medicine, Creighton University, Omaha, NE 68178, USA; ^2^Division of Cardiology, Creighton University, Omaha, NE 68178, USA

## Abstract

Takotsubo cardiomyopathy (TCM) is a unique cardiomyopathy characterized by chest pain, ECG, and regional wall motion abnormalities closely mimicking acute myocardial infarction, in the absence of significant coronary artery disease. Classic ECG changes of TCM include ST elevation or T wave inversion. However, ECG abnormalities of TCM in patients with paced ventricular rhythms have not been well characterized. Herein, we report the case of an 85-year-old pacemaker dependant female who was diagnosed with TCM four weeks following the demise of her husband. Abnormal negative T wave concordance in precordial leads and QT interval prolongation were the only new ECG findings and these reverted back to baseline on followup.

## 1. Introduction

 Takotsubo cardiomyopathy (TCM) or stress-induced cardiomyopathy is a well-known clinical entity characterized by clinical features closely mimicking acute coronary syndrome. However, the impact of underlying paced ventricular rhythm on the clinical presentation especially electrocardiogram (ECG) findings of TCM is unclear. Herein, we describe the clinical features and distinctive ECG findings of Takotsubo cardiomyopathy in a patient with paced ventricular rhythm.

## 2. Case Presentation

**Figure 1:**
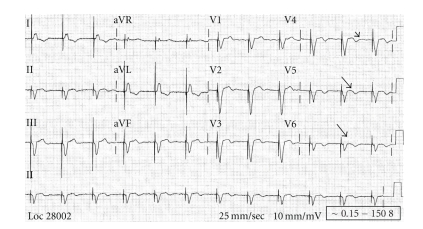
ECG at presentation: ventricular-paced rhythm at the rate of 70, prolonged corrected QT interval (QTc) of 510 milliseconds (msec) and concordant T wave inversion in lateral precordial leads.

**Figure 2:**
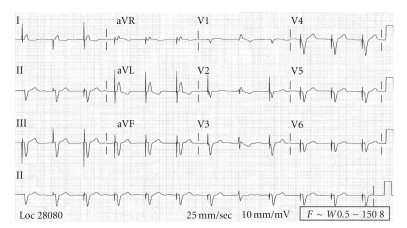
Baseline ECG (two months prior to presentation): ventricular-paced rhythm at the rate of 70, corrected QT interval (QTc) of 410 milliseconds (msec).

 An 85-year-old female presented to the emergency department with moderately severe retrosternal chest pain of 2-hour duration, 4 weeks after the demise of her husband. Pain was associated with lightheadedness, nausea, and one episode of vomiting. Medical history was significant for hypertension, diabetes mellitus, hypothyroidism, and chronic atrial fibrillation. Due to difficulty in the control of ventricular rates, she underwent atrioventricular node ablation followed by placement of a permanent pacemaker (VVIR mode) two years previously. Her medications included aspirin, atorvastatin, levothyroxine, metoprolol succinate, metformin, and dabigatran (factor Xa inhibitor — recruited as part of a research trial). On examination, blood pressure was 180/70 mmHg, heart rate: 70/min, and respirations were 18/minute. Cardiovascular examination was significant for grade 3/6 systolic murmur at apex and lung fields were clear to auscultation. ECG on admission (Figure 1) showed ventricular-paced rhythm with negatively concordant T wave inversions in left precordial leads and QTc of 510 msec. There was a non-significant increase in discordant ST segment elevation in V_2_-V_3 _ compared to baseline ECG. Her baseline ECG (2 months prior) showed a QTc of 410 msec and normal discordant T waves in precordial leads (Figure 2). A presumptive diagnosis of unstable angina was made. Blood cell count and metabolic panel were normal and troponin was borderline elevated. Echocardiogram showed an ejection fraction (EF) of 35–40% with severe apical hypokinesis and mild mitral regurgitation. There was no evidence of left ventricular outflow tract obstruction. Echocardiogram done three months prior had shown an EF of 55–60% without regional wall motion abnormalities. Emergent cardiac catheterization revealed nonobstructive coronary artery disease and ventriculogram revealed akinetic apex with normal basal segment motion, consistent with TCM. She was started on lasix and lisinopril; betablocker, oral anticoagulation, and other home medications were resumed. On follow up a month later, she was asymptomatic, and repeat echocardiogram showed an EF of 60–65% with resolution of regional wall motion abnormalities. ECG returned to baseline with normal discordant T waves in precordial leads and a QTc of 430 msec.

## 3. Discussion

 TCM is a reversible cardiomyopathy first described by Japanese physicians and named after the Japanese word for the octopus trapping pod [1]. Clinical features and ECG findings are misleadingly consistent with acute coronary syndrome. Echocardiogram typically shows akinesis of the apical and mid segments of left ventricle and normal to hyperdynamic function in the basal segments. However, TCM variants with atypical contractile patterns that selectively involve basal or mid segments and spare the apex and those that affect the right ventricle are known [2, 3]. Cardiac catheterization reveals normal or nonobstructive coronaries. Currently, the criteria proposed by the Mayo clinic group are widely used for the diagnosis of TCM [2]. About a third of patients with TCM have ST segment elevation and another third have T wave inversions. ECG is normal or shows minor nonspecific changes in the remaining [4, 5]. However, ECG changes in patients of TCM with preexisting left bundle branch block or paced ventricular rhythm are unclear and have not been systematically studied. Though certain abnormalities like discordant ST segment elevation ≥5 mm and negatively concordant ST segment depression or T wave inversion have some utility, the fallacies of identifying ischemia or infarction in patients with left bundle branch block or paced ventricular rhythm are well known [6]. In an analogous fashion, the usual ECG features of TCM are likely to be masked in the setting of paced ventricular rhythm. At presentation, our patient's ECG showed ventricular-paced rhythm with prolongation of QTc and new concordant T wave inversions. On follow up, the QTc interval and T waves normalized in parallel with clinical and echocardiographic resolution. We propose that these subtle changes in QTc and T waves might be the only recognizable changes in patients with paced ventricular rhythms developing TCM. Upto a third of patients with TCM have QTc prolongation at presentation [4]; whether this is more frequent in TCM patients with paced rhythms is unknown and needs evaluation.

 Catecholamine surge due to intense physical or emotional stress is believed to be the major pathogenic factor in TCM [1–3]. Plasma catecholamine levels are 2-3 times higher in patients with TCM as compared with those with myocardial infraction [7]. Proposed mechanisms of catecholamine-mediated injury include epicardial spasm, microvascular dysfunction [3, 8], and direct toxic effects on myocytes through an increase in intracellular calcium and oxygen-free radicals. Notably, right ventricular pacing is associated with increased sympathetic activity due to hemodynamic alterations like loss of AV synchrony, decrease in cardiac output, arterial pressure, and resultant baroreceptor reflex-mediated increase in sympathetic outflow [9]. In prior studies, plasma concentrations of catecholamines at rest and during exercise have been shown to be higher in patients with right ventricular pacing [10]. However, there is so far no evidence to suggest that patients with pacemakers are at increased risk of TCM. Treatment of TCM is generally supportive and includes diuretics, ACE inhibitors, aspirin, and beta-blockers [2, 3]. Overall, TCM carries a good prognosis with left ventricle function and apical wall motion returning to normal within days to weeks, as in our patient. However, rare reports of severe complications like cardiogenic shock needing mechanical circulatory support and death due to cardiac rupture exist [3].

 To summarize, the characteristic ECG changes of TCM may be difficult to identify in patients with paced ventricular rhythm. Subtle changes in QTc and T waves may be the only suggestive clues. The incidence, ECG abnormalities, and clinical course of TCM in patients with pacemakers are unknown and need further evaluation. In addition, future trials should try to identify if patients with pacemakers are at increased risk for TCM and define the effect of the various pacing modalities on the incidence of and outcomes in TCM.
